# Visual Aesthetics (VA) Methodology: A Strategic Approach to Facial Rejuvenation

**DOI:** 10.1111/jocd.70593

**Published:** 2025-12-15

**Authors:** Vorapot Siramangkhalanon

**Affiliations:** ^1^ Hertitude Clinic Bangkok Thailand

**Keywords:** dermal fillers, facial rejuvenation, injection safety, treatment sequencing, VA methodology, visual impact analysis

## Abstract

**Background:**

The demand for natural, harmonized facial rejuvenation has shifted aesthetics practice from localized correction toward comprehensive full‐face strategies. However, many current injection techniques remain region‐based, leading to inconsistent results and limited reproducibility. Reliable and reproducible methodologies are essential for delivering outcomes that align with patient expectations and broader aesthetic ideals.

**Objective:**

To introduce the Visual Aesthetics (VA) methodology, a structured, patient‐centered framework designed to enhance standardization, safety, and clinical outcomes in injectable facial rejuvenation.

**Methods:**

VA methodology is built on four sequential pillars across the patient journey: (1) *Communication*—eliciting true patient insight beyond stated complaints; (2) *Analysis*—applying Visual Impact Analysis (VIA) to assess proportion, framework, sagginess, and projection; (3) *Planning*—sequencing treatment via vector aesthetics (lifting → projection → volume optimization → detail enhancement); and (4) *Execution*—performing anatomy‐based targeted injections (TIs) codified with seven parameters for enhanced reproducibility and safety. Two illustrative cases (31‐ and 60‐year‐old Thai females) demonstrated its clinical application and outcome durability of this methodology.

**Results:**

Sustained aesthetic improvement was observed at 24 and 27 months in the two illustrative cases, including enhanced midface support, chin projection, and correction of tear troughs or jowl sagging. Patients reported high satisfaction. No major complications were encountered when safety protocols were followed, including adherence to vascular alert mapping and preparedness to administer hyaluronidase in the event of vascular compromise.

**Conclusion:**

VA methodology provides a reproducible and holistic framework that integrates anatomical precision with individualized goals. Further multicenter studies are warranted to validate long‐term outcomes and to explore their applicability across diverse clinical and cultural settings.

## 1 Introduction

In recent decades, the pursuit of facial rejuvenation has shifted decisively toward nonsurgical and minimally invasive procedures. Dermal fillers have become widely adopted due to their convenience, reduced downtime, and immediate aesthetic improvements [1, 2]. However, this growing popularity also raises the need for a more structured, safety‐oriented, and outcome‐driven approach to injectable treatments [3].

While product innovation has advanced significantly, clinical success still varies based on injection technique, anatomical understanding, and treatment planning [4, 5]. A key challenge is the lack of standardization in how aesthetic concerns are assessed and treated. Many practitioners continue to rely on area‐based injection strategies, addressing individual folds or volume‐deficient zones in isolation. Although effective in the short term, this approach often overlooks facial proportions, emotional expression, and the dynamic interplay of anatomical layers—leading to inconsistent or unnatural results [6].

Several methodological frameworks have been proposed to improve reproducibility, yet these remain limited by their regional focus or product‐specific orientation [7, 8]. Furthermore, global differences in anatomy and beauty perception—such as the need for projection in Asian patients versus perioral refinement in Western populations—highlight the importance of cultural adaptation in treatment design [9, 10]. The visual aesthetics (VA) methodology offers a standardized yet flexible methodology that can be applied across diverse patient groups while prioritizing safety, reproducibility, and aesthetic harmony [3, 7, 8].

In response to these limitations, the VA methodology was developed as a comprehensive, reliable, and adaptable clinical framework. It moves beyond region‐specific correction and emphasizes holistic facial evaluation, elicitation of true patient insight, and structured treatment planning. Through a four‐phase system—communication, analysis, planning, and execution—the methodology guides practitioners to deliver safer, more reproducible outcomes while respecting each patient's unique anatomy and treatment goals. Treatments are organized across multiple sessions, prioritizing high‐impact corrections at each stage to ensure both visible improvement and long‐term sustainability of results.

This review outlines the principles and structure of the VA methodology, comparing its strategic design to conventional injection paradigms and highlighting its utility across clinical, educational, and multicultural settings.

## 2 Methodology

The visual impact analysis (VIA) and target injections (TIs) were developed and refined under the clinical experience of the lead author (Dr. Vorapot Siramangkhalanon), who has applied hyaluronic acid filler with XTR technology (commercially available as Definisse, Italy [11, 12]), for over 3 years across diverse patient groups. In this clinical practice, three distinct rheological formulations were employed and aligned with the respective treatment phases: a High G' (Definisse Core—DC) filler for structural support, a Medium G' filler (Definisse Restore—DR) for volume optimization, and a Low G' (Definisse Touch—DT) filler for fine detail enhancement.

VIA was conceptualized as a structured diagnostic tool to translate aesthetic disharmony into clinical priorities. It was derived from repeated clinical observation, photographic analysis, and correlation with patient‐reported concerns, focusing on four high‐impact domains: facial proportion, framework, sagginess, and projection. Validation occurred through iterative application in more than 400 clinical cases, where consistent prioritization patterns emerged and were reproducible across practitioners trained in Thailand under this framework.

The TI system was established to enhance reproducibility and safety in filler delivery. Each TI was defined as a predetermined anatomical site, characterized by seven standardized parameters; [1] anatomical unit [2] injection plane [3] filler type [4] injection tool [5] technique [6] recommended and maximum volume, and [7] alert structures. These parameters were informed by cadaveric anatomical studies [6], clinical experience, and consensus recommendations from existing safety guidelines [7, 8]. The TI system was further validated through long‐term patient follow‐up, demonstrating durable outcomes with minimized complication rates. While this paper illustrates validation through representative case studies, future work will expand to systematic data collection with standardized outcome measures.

A safety protocol is integral to VA Methodology. Each TI incorporates recommended and maximum injection volumes to reduce the risk of overcorrection. These are based on the lead author's clinical experience and should be adapted to the individual patient's anatomy and treatment goals. Vascular alert zones are explicitly mapped, and injector choice (needle vs. cannula) is determined according to the risk profile of each site. Practitioners are advised to maintain hyaluronidase availability at all times, particularly for managing suspected vascular compromise. Early recognition protocols (e.g., blanching, pain, livedo) and stepwise intervention—including immediate filler dissolution and supportive measures—are recommended in line with published guidelines for high‐dose hyaluronidase administration [13, 14, 15, 16].

Together, VIA and TIs provide a reproducible structure for facial analysis and filler delivery, balancing clinical creativity with anatomical precision and safety.

### 2.1 Conceptual Framework of VA Methodology

The VA methodology was developed and refined through the clinical experience of the lead author (Dr. Vorapot Siramangkhalanon), who has utilized hyaluronic acid filler with XTR technology (commercially available as Definisse, Italy [11, 12]), for more than 3 years in diverse patient populations. It was conceived in response to the increasing demand for a standardized yet adaptable injectable strategy that emphasizes both aesthetic harmony and procedural safety. Grounded in extensive clinical practice and progressively refined through application, the VA methodology provides a comprehensive framework that systematically guides practitioners from patient consultation to treatment execution with precision and consistency.

### 2.2 Foundational Philosophy and Four Core Pillars

The methodology is built upon four interdependent pillars: communication, analysis, planning, and execution.

- **Communication** focuses on uncovering the patient's true insight, motivations, expectations, and barriers. While patients often present with specific complaints—such as tear troughs or nasolabial folds—these rarely reflect their true goals [17]. For instance, a patient concerned with tear troughs may actually wish to look more refreshed and less tired. Another may fixate on nasolabial folds when their deeper concern is looking younger or less saggy. This step ensures alignment between patient expression and underlying intent, laying the foundation for satisfaction and compliance.
- **Analysis** involves systematic evaluation through VIA, which was developed from repeated clinical observation, photographic analysis, and correlation with patient‐reported concerns. VIA translates aesthetic disharmony into clinical priorities by combining anatomical assessment with the psychological perception of facial messages (e.g., tiredness, sadness, fatigue). The framework focuses on four diagnostic domains:
  1. Facial proportion—assessing vertical thirds and cervico‐mental profile for balance [18].
  2. Facial framework—evaluating skeletal contour and gender‐specific ideals (e.g., oval feminine face, angular masculine jawline) [6].
  3. Sagginess—identifying gravity‐induced inversion of the “triangle of youth” [6].
  4. Projection—analyzing cephalometric parameters such as the Ricketts E‐line and facial profile angle to align the forehead, nose, lips, and chin [18].

Validation of VIA occurred through iterative application in more than 400 patients over 3 years, where consistent prioritization patterns emerged, and reproducibility was confirmed across practitioners trained under this system in Thailand.

- **Planning** synthesizes diagnostic insights into a structured roadmap. Treatment is sequenced for safety and efficacy: lateral lifting vectors → projection and elongation → volume optimization → detail enhancement. This sequence is guided by the principles of Vector Aesthetics, a directional philosophy mapping filler strategy according to structural support and visual impact.
- **Execution** refers to the clinical application of the plan using TIs. TIs were defined to improve reproducibility and safety, based on cumulative clinical experience, cadaveric validation, and risk‐mapping of vascular danger zones [6, 15, 16]. Each TI is characterized by seven parameters:
  1. Anatomical unit
  2. Injection plane
  3. Filler type
  4. Injection tool
  5. Technique
  6. Recommended and maximum volume
  7. Alert structures

These parameters, codified into a TI atlas, convert injection practice from subjective artistry into reproducible, anatomy‐informed decision‐making [19]. Long‐term patient follow‐up has demonstrated durable outcomes with minimized complication rates, although larger multicenter validation is warranted.

### 2.3 Safety and Anatomical Respect

A safety protocol is integral to VA methodology. High‐risk areas are identified in the TI atlas with vascular alert structures and maximum recommended volumes to reduce the risk of overcorrection or vascular compromise. Injection depth and tool choice (needle vs. cannula) are defined according to the risk profile of each site. Practitioners are advised to maintain hyaluronidase availability at all times, in line with published consensus on vascular complication management [13, 14, 15, 16, 20]. Early recognition of signs—such as blanching, pain, or livedo—is emphasized, with immediate intervention protocols including high‐dose pulsed hyaluronidase administration, massage, and supportive measures. This safety‐first approach reduces the risk of vision‐threatening and ischemic complications while reinforcing reproducibility and patient trust.

Together, VIA, Vector Aesthetics, and TIs form the structural and procedural backbone of VA Methodology, enabling clinicians to integrate anatomical precision with individualized treatment planning for reproducible, safe, and culturally adaptable results.

### 2.4 Treatment Planning Process

Effective treatment planning is at the heart of the VA Methodology. It transforms diagnostic insight into actionable sequences that honor both anatomy and patient intention. Unlike conventional area‐based filler approaches that treat isolated zones, the VA Methodology employs a top‐down logic that enhances structure, projection, volume, and detail in a systematic, reproducible manner [3].

#### 2.4.1 Stepwise Sequencing: Lifting → Projection → Volume → Detail

Treatment is performed following a carefully prioritized sequence:

- **Lateral (Lifting) vectors**—Structural repositioning begins with lateral vectors that support the mid‐ and lower face. This foundation enhances tissue tension and upward orientation to the soft‐tissue inferior to the vector, mitigating sagging and creating a frame for further enhancement [4].
  1. Temporal vector
  2. Zygomatic vector
  3. Subzygomatic vector
  4. Jawline vector
- **Projection and elongation**—Once lift is achieved, anterior projection and facial lengthening are addressed. Enhancing the cheek, chin, and jawline at this stage allows profile balancing and structural reinforcement.
  1. Zygomatic projection
  2. Maxilla projection
  3. Mandible projection
  4. Chin projection and elongation
- **Volume optimization**—This phase targets areas of deflation or hollowing, including deep malar fat pads, SOOF, submalar regions, and prejowl sulci. Restoration of volume is performed conservatively and harmonized with the existing lift and projection [21].
- **Detail enhancement**—The final step refines superficial elements such as tear troughs, nasolabial folds, marionette lines, and lips. This step should not be performed prematurely, as foundational correction may already improve these zones.

This sequencing avoids the common pitfall of overtreating superficial concerns without addressing structural deficiencies first [3].

### 2.5 Customization by Ethnicity and Facial Morphotype

While the sequencing of treatment remains consistent, individualization is fundamental. Patients of different ethnicities present with diverse anatomical baselines, facial proportions, and aesthetic ideals [9, 10]. For instance:

Asian faces may benefit from more anterior projection and chin elongation [9, 10].

Western faces may require a midface lift and perioral refinement [22].

Middle Eastern and Indian patients may emphasize periorbital regions [23].

VA Methodology allows adaptation of product choice, volume distribution, and treatment focus according to these variables—ensuring results are both globally applicable and culturally appropriate.

## 3 Results

### 3.1 Case Illustration 1: Visual Aesthetics Methodology in Practice

A 31‐year‐old Thai female presented with complaints of “eye bags” and “short chin,” describing herself as “looking tired” and having a “short and round face.” VIA revealed three dominant high‐impact problems: (1) eye bags, (2) short/dimpled chin, and (3) downturn lip corners.

Following the VA protocol:

**Session I: eye bags**

- **Lifting:** Temporal and zygomatic vectors were applied using *High G' filler* to elevate midface structures and improve under‐eye support.
- **Projection and elongation**: Maxilla projection (MXP2) was performed with *High G' filler* to reinforce the canine fossa and enhance midfacial anterior projection.
- **Volume optimization:** SOOF (suborbicularis oculi fat) restoration was achieved using *Medium G' filler* to address under‐eye hollowing and improve the lid–cheek junction.
- **Detail enhancement:** Tear trough refinement was completed using *Low G' filler* via microdroplet technique for precision correction.

*Total volume used: 5 cc*.

**Session II: short/dimpled chin**

- **Lifting:** Subzygomatic vector was injected with *High G' filler* to lift the lower face and support the mandibular contour.
- **Projection and elongation:** Chin elongation and mandible projection were emphasized using *High G' filler* to balance lower facial proportions and reduce roundness.
- **Volume optimization:** Not applicable in this session.
- **Detail enhancement:** Not applicable.

*Total volume used: 3 cc (lower face)*.

**Session III: Downturn lip corners**.

**FIGURE 1:**
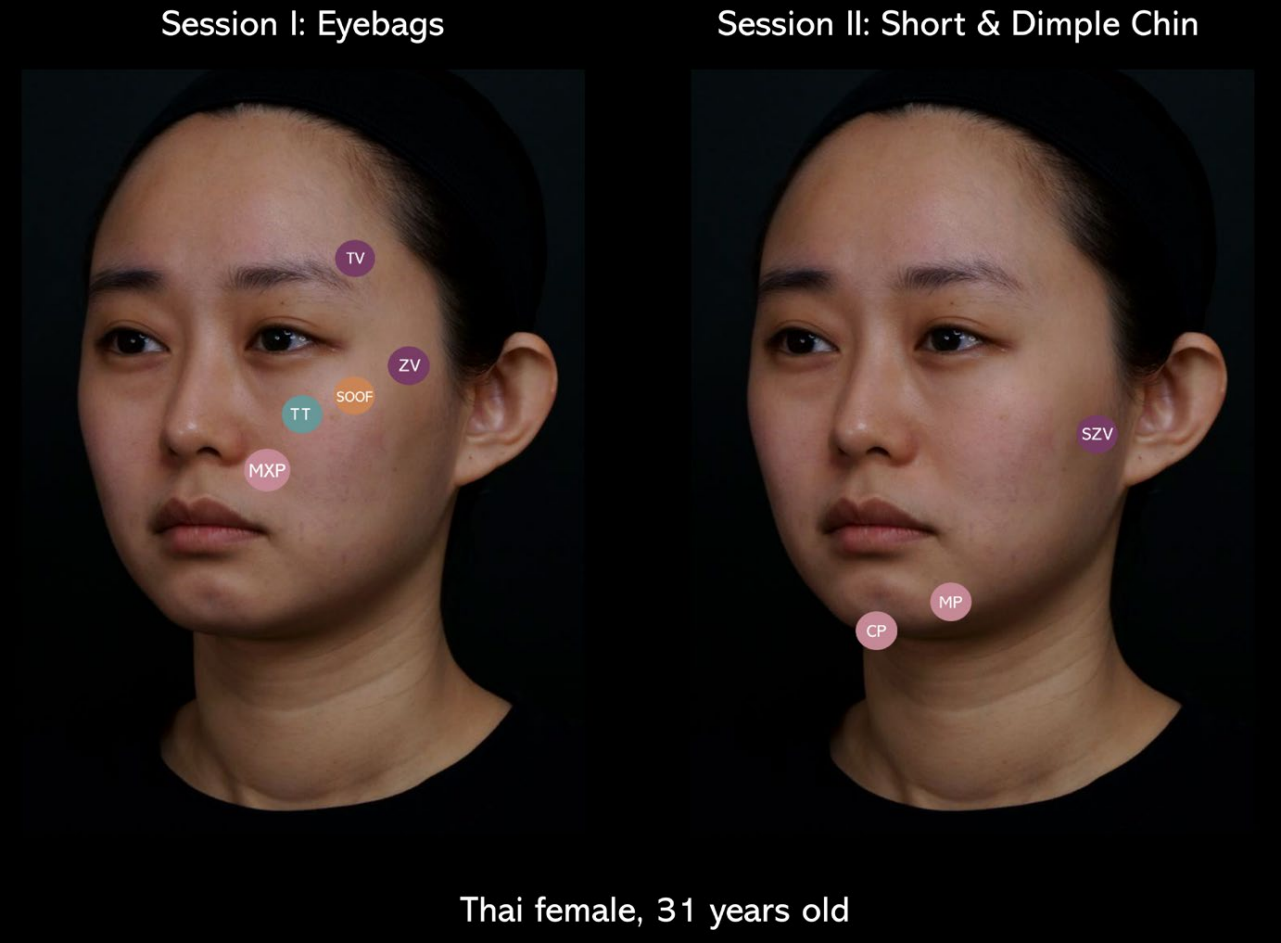
Case 1, posttreatment photographs, sessions I and II. CP, chin projection; MP, mandible projection; MXP, maxilla projection; SOOF, suborbicularis oculi fat; SZV, subzygomatic vector; TT, tear trough; TV, temporal vector; ZP, zygomatic projection; ZV, zygomatic vector.

Following strategic corrections from Sessions I and II, the downturn of the lip corners improved without direct intervention. No additional filler was required for this concern (Figure 1).

**FIGURE 2:**
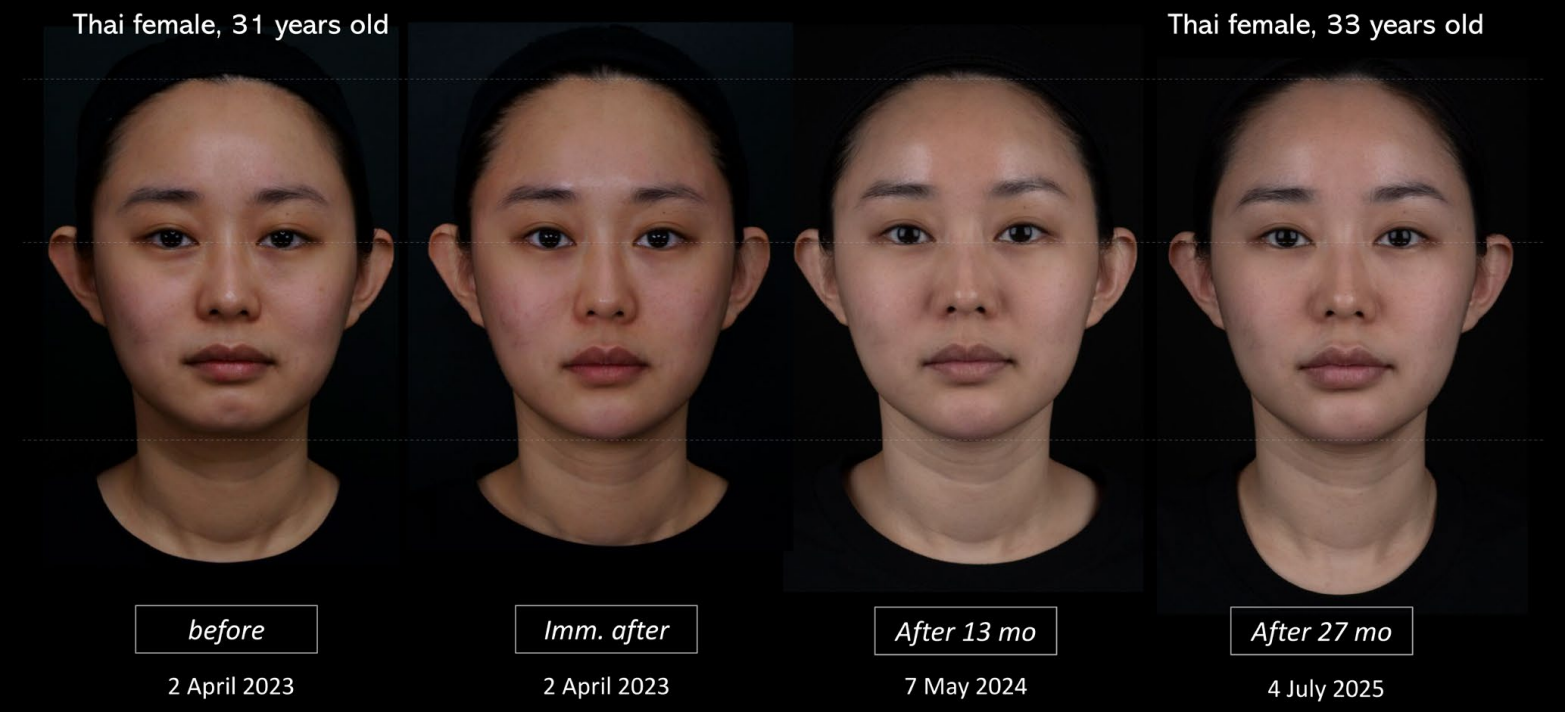
Follow‐up of case 1.

The total volume used was 5 cc in the midface and 3 cc in the lower face. Results were long‐lasting, with visible improvement sustained up to 27 months posttreatment to date (Figure 2).

This case highlights the flexibility and diagnostic depth of VA Methodology: rather than reacting to localized complaints, it maps underlying aesthetic disharmony and translates it into a safe, personalized treatment plan with durable results.

Importantly, this treatment utilized hyaluronic acid fillers developed with XTR (eXcellent Three‐dimensional Reticulation) technology [11, 12], known for their structural integrity, moldability, and longevity. Clinical observations and follow‐up of this patient at 13 and 19 months posttreatment demonstrated sustained aesthetic improvement without significant adverse effects—underscoring both the long‐term safety and efficacy of the filler material when applied within a strategic framework like VA Methodology.

### 3.2 Case Illustration 2: Application of VA Methodology in an Older Age Group

A 60‐year‐old Thai female presented with the concern that she looked older than she felt. Her complaints included nasolabial folds and facial sagging. Upon deeper communication, her true insight was a desire to “age gracefully,” without aiming to look significantly younger than her age, but rather to appear less droopy and more uplifted.

VIA revealed the following high‐impact concerns:

1. Jowl sagginess
2. Eyebags and tear troughs
3. Poor lip projection

Following the VA methodology:

**Session I—Lower face rejuvenation: Jowl sagginess**

- **Lifting**: Zygomatic, subzygomatic, and jawline vectors were treated with *High G' filler* to re‐establish structural integrity in the mid and lower face.
- **Projection and elongation**: Mandibular and chin projection were restored using *High G' filler* to improve jawline definition and vertical balance.
- **Volume optimization**: The prejowl sulcus was corrected with *Medium G' filler* to smooth the mandibular contour.
- **Detail enhancement**: Not applicable.

**Session II—Midface rejuvenation: Eyebags and tear troughs**

- **Lifting**: Temporal vector was reinforced (zygomatic vector had been previously treated in Session I).
- **Projection and elongation**: Anterior cheek projection was addressed to support the lid‐cheek junction using *Core*.
- **Volume optimization**: Volume loss in the deep medial cheek fat pads and SOOF was corrected using *High G' filler* and *Medium G' filler*.
- **Detail enhancement**: Tear troughs were treated using *Low G' filler* via microdroplet injection for refinement.

**Session III—Perioral rejuvenation: Lip projection**

**FIGURE 3:**
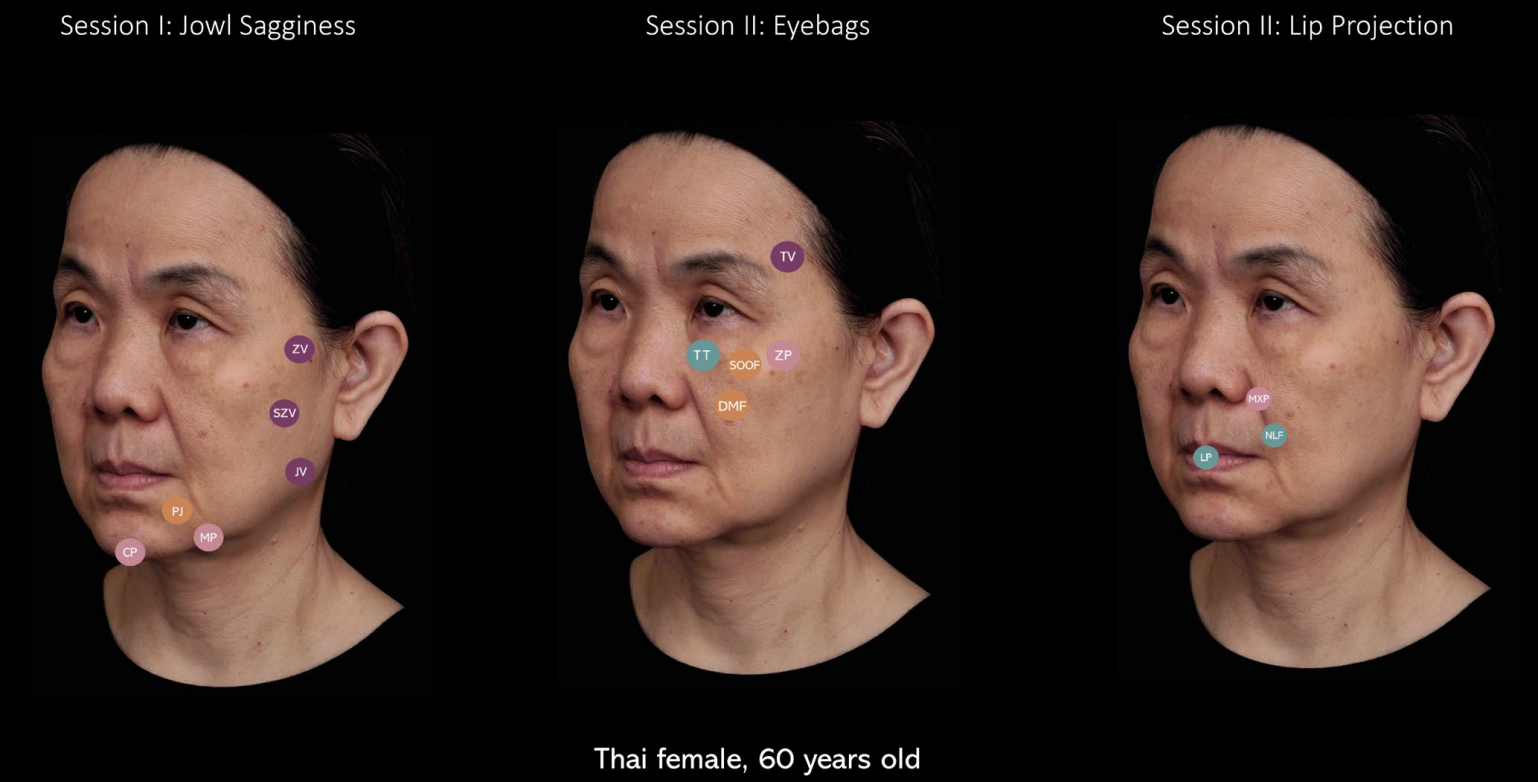
Case 2, posttreatment photographs, sessions I and II. CP, chin projection; DMF, deep medial fat pads; JV, jawline vector; MP, mandible projection; PJ, prejowl sulci; SZV, subzygomatic vector; TT, tear trough; TV, temporal vector; ZP, zygomatic projection; ZV, zygomatic vector.

- **Lifting**: Zygomatic vectors were already optimized in Session I.
- **Projection and elongation**: Maxilla projection was performed with *Core* to support upper lip projection.
- **Volume optimization**: Not applicable.
- **Detail enhancement**: The perioral region—including nasolabial folds and lips—was treated using *Low G' filler* to enhance projection (Figure 3).

**FIGURE 4:**
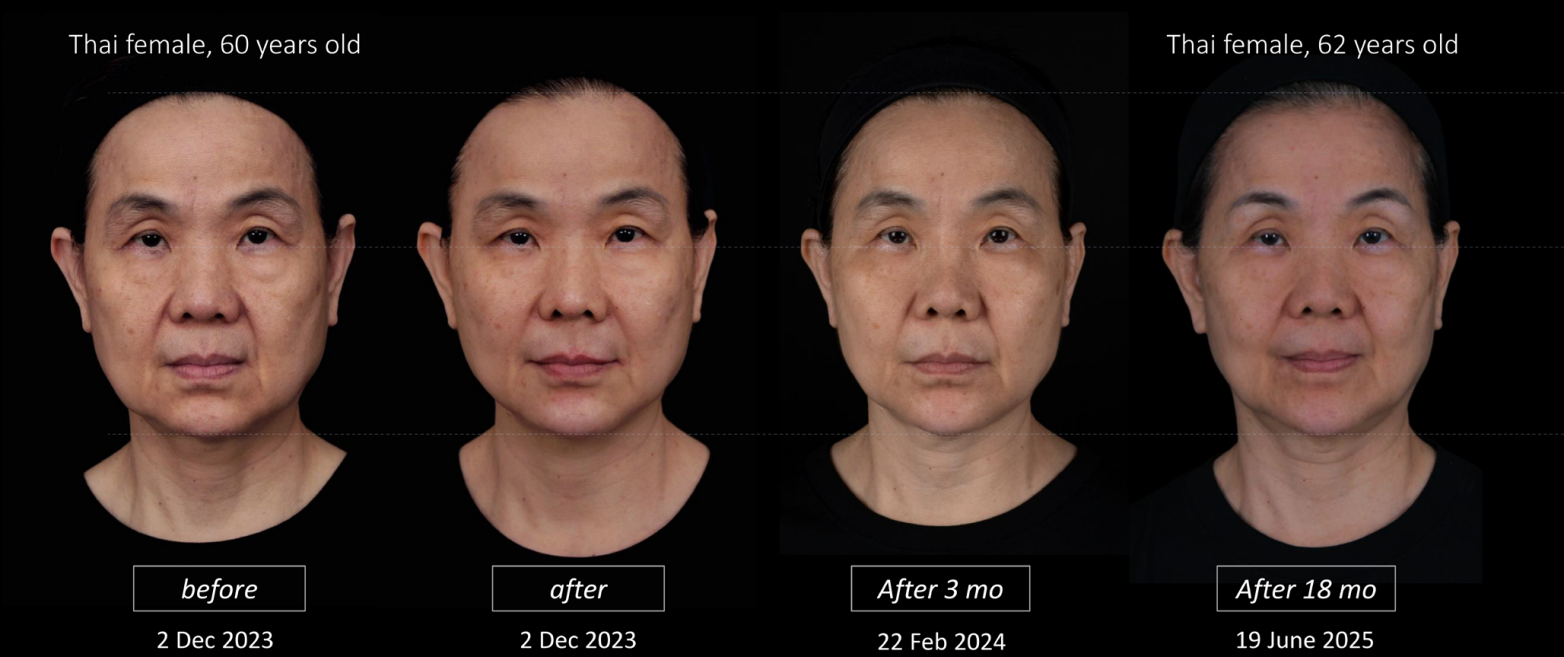
Follow‐up of case 2.

Total volume used was 7 cc in the midface and 5 cc in the lower face. The patient reported high satisfaction, noting she looked more rested and lifted, while maintaining her natural identity. Clinical follow‐up at 3 months and 18 months confirmed long‐term effectiveness and safety (Figure 4).

This case reinforces the adaptability of VA methodology across age groups and aesthetic goals, enabling customized treatment aligned with both anatomical needs and emotional intent.

### 3.3 Outcomes and Safety

The VA methodology has been applied in routine clinical practice across a large number of patients over the past 3 years. For this manuscript, two representative cases are illustrated, supported by descriptive observations from our wider patient cohort. In our practice, most patients treated with the VA methodology were female and of Asian ethnicity, reflecting the demographic profile of our clinical population, although patients of diverse age groups and ethnic backgrounds were also included.

**Patient satisfaction** was consistently high, as reported during routine follow‐up visits. Many patients described improvements that went beyond correction of isolated concerns, instead noting enhanced freshness, balance, and harmony of the overall face. Photographic documentation likewise demonstrated reproducible improvements in total facial appearance rather than region‐specific changes. While validated tools such as GAIS or FACE‐Q were not systematically collected, these will be incorporated in future prospective, IRB‐approved studies.

### 3.4 Follow‐Up

Improvements were consistently observed in the short term, and in several patients with available long‐term follow‐up, outcomes were maintained for more than 2 years without the need for secondary correction.

### 3.5 Safety

The safety profile of the VA Methodology was favorable. Adverse events were limited to transient swelling and bruising, which resolved spontaneously within days, consistent with the known profile of hyaluronic acid filler injections. No cases of vascular occlusion, skin necrosis, or vision‐threatening complications were encountered in our practice when safety protocols—including vascular mapping, recommended volume limits, and hyaluronidase preparedness—were followed.

### 3.6 Limitations

The outcomes described here are based on patient‐reported satisfaction and photographic documentation rather than systematically validated scales. Although this provides meaningful real‐world insights, future prospective, multicenter studies with standardized tools (e.g., GAIS, FACE‐Q) and formal complication tracking are necessary to further validate the reproducibility and generalizability of the VA Methodology.

## 4 Discussion

The VA methodology was developed to address the limitations of conventional region‐based filler techniques, which often treat isolated concerns without fully considering facial proportions, balance, or patient perception. By emphasizing communication, structured analysis through VIA, and stepwise sequencing of lifting, projection, volume, and detail, the methodology provides clinicians with a reproducible framework that integrates anatomy with patient goals.

Several structured frameworks have been proposed to improve reproducibility in aesthetic medicine, most notably MD Codes, which have become widely adopted worldwide [3]. MD Codes integrate the concept of Emotional Attributes and employ the MD ASA (Multi‐Dimensional Aesthetic Scan Assessment) to translate facial messages into treatment priorities. Using the foundation–contour–refinement (FCR) principle, it provides a systematic and reproducible approach, with treatment often organized into structured sessions. This has contributed significantly to global injector education by creating a common language for planning and communication.

The VA Methodology shares the goal of standardization but offers a different perspective. Rather than starting from emotional attributes, it begins with uncovering patient motivation, insight, expectations, and barriers, ensuring alignment between clinical treatment and personal goals. VIA then identifies high‐impact problems most strongly linked to patient insight, and treatments are sequenced so that each session delivers a visible and meaningful impact. Volume planning is guided by recommended and maximum levels per TI, while remaining flexible to accommodate anatomical variation.

These distinctions illustrate that VA Methodology and MD Codes are not competing but complementary approaches. While MD Codes provide a highly structured and globally recognized algorithm, VA Methodology offers a flexible, insight‐driven strategy that prioritizes high‐impact outcomes session by session. Together, they represent the evolution of structured aesthetic practice, offering injectors multiple paradigms to enhance communication, reproducibility, and patient satisfaction.

A core principle of VA Methodology is safety. Each TI incorporates defined anatomical planes, alert structures, and volume recommendations. Hyaluronidase availability and vascular safety protocols are mandatory, consistent with recent evidence supporting its role in managing embolic complications [14].

In summary, VA Methodology contributes a holistic and adaptable framework that complements established systems such as MD Codes. Its focus on communication, high‐impact outcomes, and integrated safety protocols adds meaningful clinical and educational value, while ongoing research and validation will further consolidate its role in advancing patient‐centered, reproducible aesthetic practice.

## 5 Conclusion and Future Perspectives

By translating complex facial analysis into structured treatment plans, VA Methodology bridges scientific precision with artistic judgment, allowing treatments that are both predictable and personalized. This holistic and adaptable insight‐driven framework provides an alternative to product‐ or area‐based injection approaches, grounding treatment in anatomical understanding and aesthetic harmony.

Future perspectives include validating the VA Methodology through longitudinal studies across diverse patient populations and ethnic groups, as well as incorporating its principles into clinical education. Such work will help to focus on refining its applicability, enhancing reproducibility, and supporting best practices in injectable facial rejuvenation (Table 1).

**TABLE 1:** Target injection summary.

Anatomical unit	Vector aesthetics	Target depth of injection	Product	Injection tool	Injection technique	Volume per side (mL)		Alert structure
						Recommend	Max	
Lateral (Lifting) vector								
Temporal vector	TV	b	DC	S	B	0.5	0.7	Superficial temporal artery, anterior deep temporal artery
		in	DC	C	F	0.5	1.0	Middle temporal vein
		sc	DC	C	B	0.5	1.0	Superficial temporal artery
Zygomatic vector	ZV	b	DC	S	B	0.1	0.3	Middle temporal artery & vein, transverse facial artery
		sub	DC	C	L	0.3	0.5	—
Sub‐zygomatic vector	SZV	sm	DC	C	F	0.5	1.0	Transverse facial artery, parotid gland & duct
Jawline vector	JV	b	DC	C	F	0.3	0.5	Masseter muscle
		sc	DC	S	B	0.5	1.0	Facial artery
Projection & elongation								
Lateral cheek projection	Z1	b	DC	S	B	0.1	0.3	Zygomaticofacial artery, infraorbital artery
Anterior cheek projection	Z2	b	DC	S	B	0.2	0.3	Zygomaticofacial artery
Inferior cheek projection	Z3	b	DC	S	B	0.2	0.3	Zygomaticofacial artery
Anterior nasal spine	MXP1	b	DC	S	B	0.3	0.5	Columellar artery
Canine fossa	MXP2	b	DC	S	B	0.3	0.5	Facial artery
Mandible projection	MP	sc	DC	C	F	0.5	1.0	
		b	DC	S	B	0.3	0.5	Submental artery
Chin projection & elongation	CP	b	DC	S	B	0.3	0.5	Mental artery
		sc	DC	C	F	0.3	0.5	Submental artery
Volume optimization								
Deep malar fat	DMF	DMF	DC	C	F	0.5	1.0	Infraorbital artery
SOOF	SOOF	SOOF	DR	C	F	0.5	1.0	Infraorbital artery
Sub‐Malar	SM	sc	DR	C	F	0.5	1.0	Facial artery
Pre‐Jowl	PJ	sc	DR	C	F	0.5	1.0	Labio‐mental artery
Detail enhancement								
Tear trough	TT	b	DT	C	M	0.5	1.0	Infraorbital artery, lower eyelids
Nasolabial folds	NL	sc	DR	C	F	0.5	1.0	Facial artery
		id	DT	S	L	0.3	0.5	Facial artery
Lips	LP	sc	DR/DT	C	L	1.0	2.0	Superior & inferior labial artery
		id	DR/DT	S	L	0.5	1.0	Superior & inferior labial artery
Marionette's lines	M	sc	DT	C	L	0.5	1.0	Labio‐mental artery
		id	DT	S	L	0.3	0.5	Labio‐mental artery

*Note:* Depth of Injection: b – Periosteum, DMF – deep malar fat pad, id – Intradermal, in – Inter‐fascia, sc – Subcutaneous, SOOF—suborbicularis oculi fat; sub—Sub superficial musculoaponeurotic system (SMAS), sm—Supra SMAS. Product: DC—High G' filler (Definisse Core), DR—Medium G' filler (Definisse Restore), DT—Low G' filler (Definisse Touch). Injection tool: C—Cannula, S—Sharp Needle. Injection technique: B—Bolus, F—Fanning, L—Linear, M—Microdroplets.

## Author Contributions

Vorapot Siramangkhalanon had the idea, developed and designed the methodology, wrote the draft manuscript, revised, and approved the final version of the manuscript.

## Funding

This study was sponsored by A. Menarini (Thailand). Medical editorial assistance were provided to author by Marc Baay (P95 Clinical and Epidemiology Serivces), and funded by A. Menarini (Thailand) Ltd. Neither honoraria nor other forms of payment were made for authorship.

## Disclosure

Vorapot Siramangkhalanon is self‐employed in the Hertitude Clinic, Bangkok, Thailand. No logistical or financial support for the execution of this work was received. Dr. Vorapot Siramangkhalanon serves as a consultant for A. Menarini (Thailand) and A. Menarini Asia‐Pacific. VA Methodology is an intellectual property belonging to the author. Some of the concepts included in this paper have been presented by the author in seminars as part of the A. Menarini (Thailand) event, and images have been included in A. Menarini's medical educational materials.

## Ethics Statement

All medical procedures described in this report were conducted in accordance with the Declaration of Helsinki. All participants were informed and provided consent to publish their photographs as part of this work. This article does not contain any studies with human participants or animals performed by the author.

## Consent

Written informed consent was obtained from all patients included in this study for the anonymized use of clinical images for publication.

## Conflicts of Interest

Vorapot Siramangkhlanon has severed as a consultant for speaking events and medical education for A. Menarini (Thailand) and A. Menarini Asia‐Pacific. Some of the concepts included in this paper have been presented by the author in seminars, and medical conferences as a part of a Menarini Medical Education and images used in this article have been included in Menarini's medical educational materials.

## Data Availability

The data that support the findings of this study are available from the corresponding author upon reasonable request.
